# Antisense Oligonucleotide against hTERT (Cantide) Inhibits Tumor Growth in an Orthotopic Primary Hepatic Lymphoma Mouse Model

**DOI:** 10.1371/journal.pone.0041467

**Published:** 2012-07-24

**Authors:** Bo Yang, Rui-li Yu, Shuai Tuo, Chao-wei Tuo, Qiu-zhen Liu, Ning Zhang, Xue-chun Lu, Xiao-hua Chi, Shu-bao Lv, Li-li Cai

**Affiliations:** 1 Department of Geriatric Hematology, Chinese PLA General Hospital, Beijing, China; 2 Institute of Otorhinolaryngology, Chinese PLA General Hospital, Beijing, China; 3 Department of Ultrastructural Pathology, Hospital of Chinese PLA, Shenyang, China; 4 Department of Pharmacy, the Second Artillery General Hospital, Beijing, China; 5 Department of Statistics, Unit of Chinese PLA, Baicheng, China; 6 Department of Geriatric Laboratory Medicine, Chinese PLA General Hospital, Beijing, China; Innsbruck Medical University, Austria

## Abstract

**Background:**

Human xenograft models, resulting from orthotopic transplantation (implantation into the anatomically correct site) of histologically intact tissue into animals, are important for investigating local tumor growth, vascular and lymphatic invasion at the primary tumor site and metastasis.

**Methodology/Principal Findings:**

We used surgical orthotopic transplantation to establish a nude mouse model of primary hepatic lymphoma (PHL), HLBL-0102. We performed orthotopic transfer of the HLBL-0102 tumor for 42 generations and characterized the tumor cells. The maintenance of PHL characteristics were supported by immunohistochemical and cytogenetic analysis. We also report the antitumor effect of Cantide, an antisense phosphorothioate oligonucleotide against hTERT, on the growth of HLBL-0102 tumors. We showed a significant, dose-dependent inhibition of tumor weight and serum LDH activity in the orthotopically transplanted animals by Cantide. Importantly, survival was prolonged in Cantide-treated HLBL-0102 tumor-bearing mice when compared to mock-treated mice.

**Conclusions/Significance:**

Our study provided the basis for the development of a clinical trial protocol to treat PHL.

## Introduction

The relevance of preclinical, animal models of cancer depends on how closely the xenografts mimic the histological, biochemical and metastatic patterns of the original tumor. Current rodent tumor models include transgenic or subcutaneously-growing human tumors in immune-deficient mice. However, the lack of metastasis from the subcutaneous site is a major limitation of subcutaneous xenografts, making it important to develop preclinical models of cancer which recapitulate invasion and metastasis [1], [2]. Interestingly, it was observed that hepatocellular carcinoma (HCC) cells did not exhibit invasion and metastasis unless they were transplanted orthotopically [1], [3]. Although orthotopic implantation has been performed with tumor cells [4] as well as histologically intact tissue [5]–[9], it has been shown that the biologic behavior of natural tumors is more closely reproduced with transplantation of histologically-intact tissue. These data suggest that human xenografts, resulting from orthotopic transplantation (implantation into the anatomically correct site) of histologically intact tissue into animals, provide an important model system to investigate local tumor growth, vascular and lymphatic invasion at the primary tumor site and metastasis. Interestingly, the chemosensitivity of orthotopically transplanted human small-cell lung carcinoma and colon cancer has been reported to differ significantly from the subcutaneously transplanted model [10], [11], suggesting that tumor microenvironment also plays an important role in modulating chemosensitivity [12], [13]. Therefor the orthotopic transplantation model may also be a useful tool to predict the *in vivo* response to specific drugs.

Although liver involvement is commonly seen in the late stages of lymphoma, primary hepatic lymphoma (PHL) is relatively rare [14], [15]. PHL typically is shown on imaging studies as a solitary mass in the liver, along with elevated lactate dehydrogenase (LDH) levels [16]. Although the pathogenesis is not clear, PHL is often seen in HBV, HCV and HIV patients [17]–[19]. PHL patients have a poor prognosis with a median survival as low as 6 months [20]. Although surgery, radiotherapy and multi-agent chemotherapy are currently used as standard treatments, the optimal therapy for PHL is still not clearly defined [20], [21].

Recent reports point to the possibility of treating tumors by gene silencing technology using antisense oligonucleotide [22]. Antisense oligonucleotide targeting cell cycle or apoptosis related genes have been used in some malignancies [23], [24]. Antisense oligonucleotides targeting Bcl-2 had promising results in a Phase I clinical trial for non-Hodgkin’s lymphoma [25]. Also, an antisense oligonucleotide against survivin inhibited tumor growth by inducing apoptosis in lung cancer cells [26].

Telomerase, a ribonucleoprotein complex that is responsible for maintaining telomeres, is activated in 90% of all cancers [27] and is considered an important target in cancer therapy. An antisense oligonucleotide targeting the human catalytic subunit of telomerase, hTERT, has been shown to inhibit proliferation and induce apoptosis [28]. One of the antisense oligonucleotides, Cantide, designed to hybridize with the 3′-untranslated sequences in human hTERT mRNA, was shown to specifically down-regulate hTERT mRNA levels, telomerase activity and trigger apoptosis in an in vitro assay using HCC cells [27]. Although Cantide has not been used in human clinical trials, it was shown to exhibit antitumor activity in nude mouse tumor xenografts [29]. Additionally, Lin et al. examined the effectiveness of Cantide in a xenograft model of HCC in mice, and they found that it reduced HCC tumor growth in a dose-dependent manner [30].

In this study, we successfully establish a human xenograft model of PHL in nude mouse (HLBL-0102) and performed detailed characterizations of the tumor cells that included comparing the proliferation and DNA indexes of the tumor cells to normal liver cells. We also evaluated the antitumor effect of Cantide on HLBL-0102 tumor xenografts *in vivo* by determining the rate of tumor inhibition and survival.

## Materials and Methods

### Animals

This study was approved by the IRB of Chinese PLA General Hospital, Beijing, China. Written informed consent was obtained from the patient before resection of hepatic non-Hodgkin’s B cell lymphomas. Male and female athymic BALB/C-nu/nu nude mice between 4 to 6 weeks of age (18–22 grams) were obtained from the National Institute for the Control of Pharmaceutical and Biological Products, Beijing, China. The animals were housed and bred in a pathogen-free, HEPA-filtered environment. Cages, food and bedding were sterilized by autoclaving. Animal diets were obtained from Beijing Laboratory Animal Research Center. All animal studies were conducted in accordance with the principles and procedures outlined in Regulations for Administration of Affairs Concerning Experimental Animals approved by the State Council on October 31, 1988 and promulgated by Decree No. 2 of the State Science and Technology Commission, China, on November 14, 1988.

### Establishment of an Orthotopic Xenograft Nude Mouse Model for Liver Lymphoma

#### Specimen preparation

In this study, we used orthotopic implantation to establish a human primary hepatic non-Hodgkin’s B cell lymphoma xenograft, HLBL-0102, in nude mice. The xenograft had been transferred for 42 generations (more than 3 years) by orthotopic passage (liver to liver) in nude mice, and exhibited 100% transplantability.

For xenograft preparation, fresh surgical specimens were obtained from liver lymphoma tissues resected from a 53-year-old man with primary hepatic non-Hodgkin’s B cell lymphoma. The patient was diagnosed according to the 2001 World Health Organization (WHO) classification of neoplastic diseases of the hematopoietic and lymphoid tissues, which was the current edition when the study was conducted. The PLL diagnosis criteria were the same in the 2001 edition as in the newer 2008 edition. The patients were classified as clinical stage IEA based on the Ann-Arbor system of classification. The patient underwent surgery in the Department of Hepatobiliary Surgery, No. 202 Hospital of People Liberation Army (PLA), Shenyang, China.

Macroscopic examination showed multiple tumor nodules in the right lobe of the liver and a large mass of 5.3 cm ×3.5 cm ×3.0 cm resulting from the fusion of multiple tumor nodules in the left lobe of the liver. Anatomical analysis of the tumor nodules revealed a grayish-white fishlike cross-section, a yellowish-white area in the middle of nodule, and red irregular hyperemia bands located at the border of nodule. There was no involvement of other tissues, organs, and distal lymph nodes. The specimen was obtained from the tumor nodule of the left lobe of the liver. Laboratory data prior to surgery showed that the patient was positive for hepatitis B surface antigen (HBsAg) and had elevated lactate dehydrogenase activity (LDH) levels (1200U/L). The patient was tested normal in liver function, alpha-fetoprotein (AFP) levels, carcinoembryonic antigen (CEA) levels and myelogram. The surgical specimen was stored in RPMI 1640 medium (Hyclone, Logan Utah, USA) supplemented with 1% penicillin and streptomycin (Sigma-Aldrich, St. Louis, MO, USA) at 4°C and was transplanted by surgical orthotopic implantation (SOI) into nude mice within 2 h after surgery.

### Surgical Orthotopic Implantation Procedure

Before implantation, specimens were washed twice with antibiotic-containing RPMI1640 medium, for at least 10 min each time, to prevent possible contamination and infection. Necrotic and non-cancerous tissues were removed from the specimen and the remaining malignant tissues were minced into small pieces of approximately 1 mm in each dimensions.

For surgical orthotopic liver implantation, nude mice were anesthetized with an intravenous injection of 0.5% sodium pentobarbital (30 mg/kg). A small transverse incision of the left superior belly was made and the liver was exteriorized. The capsule of the left lobe of the liver was removed and two pieces of 1-mm^3^ tumor tissues per mouse were implanted. The small tumor tissues were sutured into the incised liver using a 10-0 surgical suture. The liver was returned to the abdominal cavity, and the abdominal wall and the skin were closed. Implanted animals were kept in a insulated facility under HEPA filtration and observed for signs of tumor growth, weight loss and distress. Moribund mice were selected and sacrificed and the transplanted hepatic tumors were harvested. The harvested tumor tissue was used for morphological, immunohistochemical, biochemical and cytogenetic analysis. Tumor growth, invasion, metastasis, and animal survival were evaluated in the remaining animals, which were maintained until they showed signs of distress or died.

### Evaluation of Growth and Metastasis

Detailed anatomical examination was performed on all the animals that were sacrificed or died naturally. Gross examination included measurements of tumor volume, tumor weight, body weight, evaluation of local infiltration. Tumor sizes were monitored with calipers. Specimens of tumor tissues, lymph nodes, and internal organs were fixed in 10% formalin and embedded in paraffin. Histological examination of tumor sections were performed by stained with hematoxylin and eosin for light microscopy.

**Figure 1:**
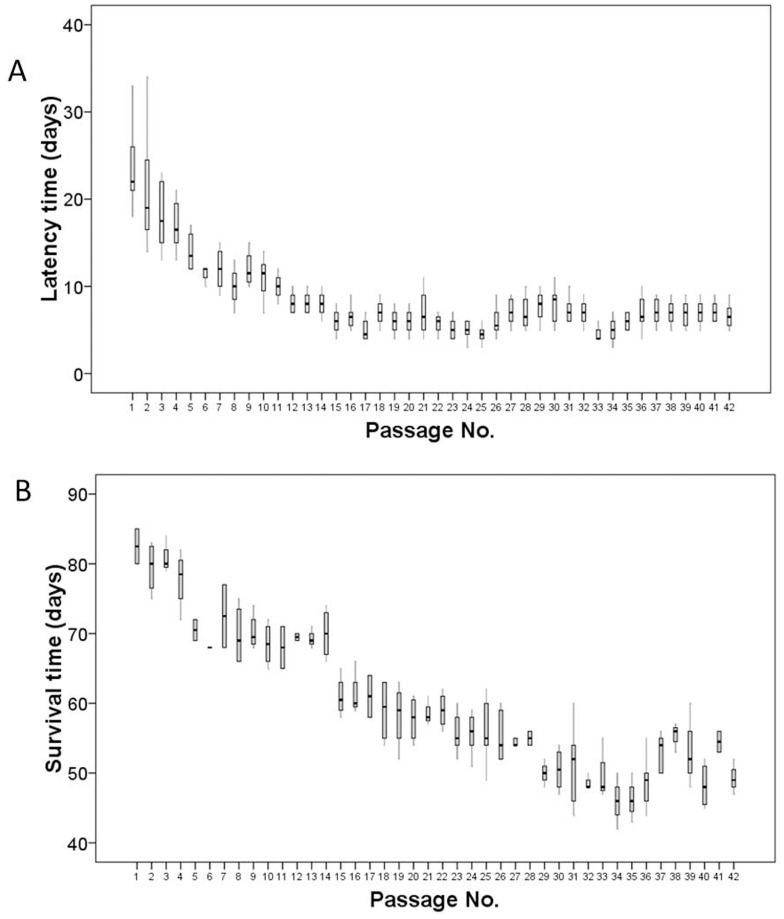
The change of latency and survival time during establishment of HLBL-0102. (A) Change in latency during establishment of HLBL-0102 model. Latency period (time from implantation until the tumor measured 0.5 cm in size) was observed until passage 42. (B) Change in survival time during establishment of HLBL-0102.

### Immunohistochemistry

We used immunohistochemistry to characterize the hepatic lymphoma used in xenograft using antibodies against CD20, CD79a, Bcl-6, CD10, and IRF4 (also known as Mum1). Briefly, antigen retrieval was performed by treating tissue sections with 0.1% trypsin for 30 minutes at RT. Endogenous peroxidase was inactivated with 3% H_2_O_2_ for 5 minutes in RT. The sections were then washed three times in TBS (pH7.6) for 5 minutes each and blocked in 5% normal serum for 30 minutes at RT. The sections were incubated with a 1∶100 dilution of primary antibody (monoclonal antibodies against human CD20 (Cat# R701301), CD79a (Cat# M705001), Bcl-6 (Cat# M721101), CD10 (Cat# M730801) and IRF4 (Cat# M725901); DAKO, USA) for 30 minutes at RT, washed and then incubated with a 1∶100 dilution of biotin-labeled rabbit anti-mouse polyclonal antibody for 30 minutes at RT. The sections were washed and incubated with a 1∶400 dilution of HRP-conjugated streptavidin for 30 minutes at RT. Color was developed by incubating with DAB for 5–10 minutes.

### Determination of Proliferation Index (PI) and DNA Index (DI)

Fresh tumor tissue was washed with PBS and cut it into 1 mm^3^ size before digesting in a 30-fold volume of 0.05% collagenase at 37°C for 60 minutes. Cells were isolated using a No. 100 mesh to remove undigested tissue. Cells were then centrifuged in 1.2K rpm for 5 minutes, washed twice in PBS and centrifuged in 800 rpm for 3 minutes to remove cell debris. The purified cells were stained with 50 µg/ml Propidium iodine (Sigma-Aldrich, St. Louis MO, USA) and the DNA content quantified in a flow cytometer (FACS-420,B.D, USA). DNA index was measured by the following formula: G_0_/G_1_ DNA content/average DNA content of normal lymphocytes. Proliferation index (PI) was measured using the following formula: PI = (G2/M _cell number_+S_cell number_)/(G2/M _cell number_+S_cell number_+G_0_/G_1cell number_)×100%.

### Oligonucleotide and Drugs

Antisense phosphorothioate oligonucleotide, Cantide (5′-ACTCACTCAGGCCTCAGACT-3′), was a kind gift from the Beijing Institute of Radiation Medicine (Beijing, China). The selection of antisense ODNs against hTERT was described previously(31). Briefly, Cantide was synthesized by an Applied Biosystems Model 391 DNA synthesizer on solid supports using Oligo Pilot II DNA (Amersham-Pharmacia, Piscataway, NJ, USA) and purified by high-performance liquid chromatography (HPLC) (Waters Delta Prep 4000, Milford, MA, USA) with SOURCE 15Q (Amersham Pharmacia, Piscataway, NJ, USA). The purity of Cantide was over 95%. The positive control was 5-Fluorouracil (5-FU), purchased from the Shanghai Donghaipu Pharmaceuticals Company (Shanghai, China) and the negative control was 0.9% saline. Cantide and 5-FU were diluted to the appropriate doses with 0.9% saline solution.

**Figure 2:**
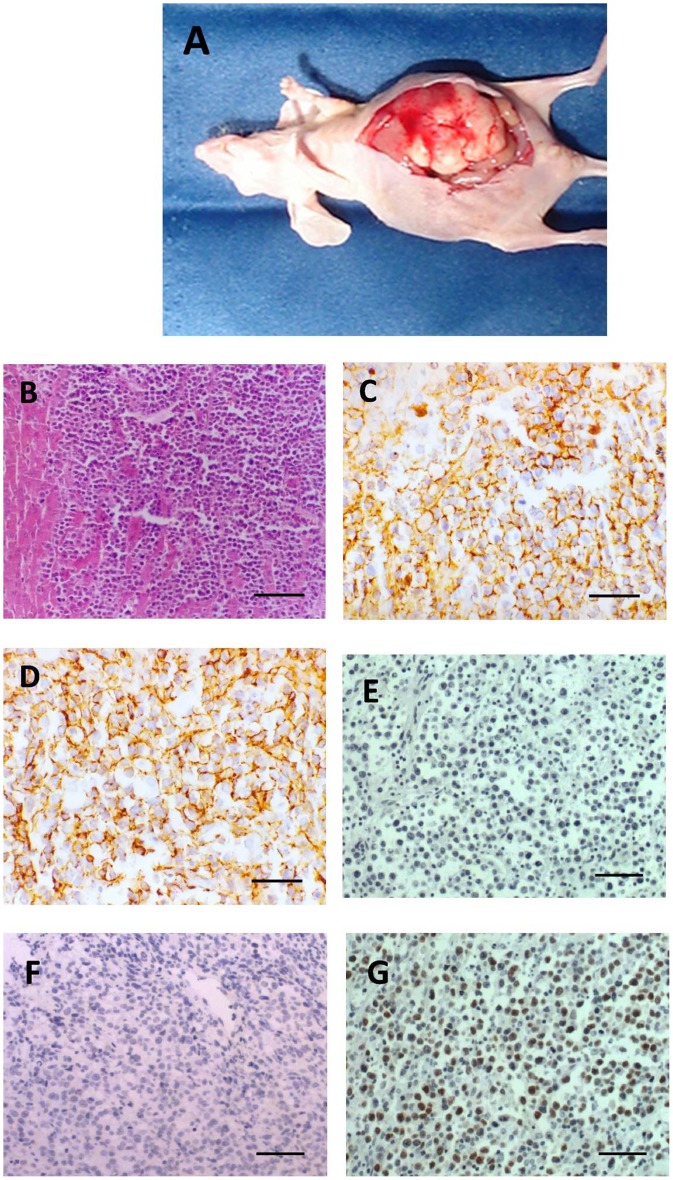
Pathological examination of HLBL-0102 model. (A) Representative image of an orthotopically transplanted mouse in the 37^th^generation. (B) Histological examination of tissue section from PHL in HLBL-0102 mice (H&E staining; 150x). (C) Immunohistochemical staining of HLBL-0102 PHL for CD20 (LSAB method; 400x). HLBL-0102 cells were strongly positive for B cell lymphoma marker, CD20. (D) Immunohistochemical staining of HLBL-0102 PHL for CD79a (LSAB method; 400x). HLBL-0102 cells were strongly positive for B cell lymphoma marker, CD79a. (E) Immunohistochemistry staining of HLBL-0102 PHL for Bcl-6 (LSAB method; 150X). (F) Immunohistochemistry staining of HLBL-0102 PHL for CD10 (LSAB method; 150X). (G) Immunohistochemistry staining of HLBL-0102 PHL for IRF4 (LSAB method; 150X). Scale bar  = 50 µM for B, E, F, G. Scale bar  = 20 µM for C and D.

### Treatment of HLBL-0102 Xenografts with Cantide and 5-FU

Each experiment was performed on day 3 after tumor transplantation. There was one untreated (vehicle control) group and 5 treatment groups (including Cantide 12.5, 25, 50, 75 mg/kg/d and 5-FU 10 mg/kg/d group) comprising 8 animals in each group. Cantide and 5-FU were administrated intravenously (i.v.) daily for 20 days by bolus infusion into the tail vein. The 8 mice in the untreated group received the same volume of physiological saline intravenously. The body weight and general physical conditions of the animals were recorded daily. After the last administration (i.e. day 24 after transplantation), the mice were sacrificed by cervical dislocation and autopsy was performed. The tumors growing on the liver were removed and weighed. The percentage of tumor growth inhibition was calculated as:

Inhibitory rate (%) =  (1−*W*_treat_/*W*_control_) ×100 where *W*_treat_ is the tumor weight of the treated group and *W*_control_ is the tumor weight of the non-treated group.

Three independent experiments were conducted and a total of 144 mice (48 mice per experiment) were used.

**Table 1:** FCM analysis.

Groups	Normal liver (n = 30)	HLBL-0102 tumor (n = 260)	Original patient tumor (n = 30)
DNA index	1.0±0.1	1.7±0.2*	1.7±0.2*
Proliferation index	14.7±1.4	34.7±2.2*	34.2±1.6*
Ploidy	euploid	aneuploid	aneuploid

* P<0.05 significantly different from normal liver, Bonferroni’s correction.

### Detection of LDH Activity

Serum LDH activity was determined using the COBAS INTEGRA LDHI2 kit purchased from Roche (Mannheim, Germany) according to the manufacturer’s instructions. LDH activity was detected using the Roche COBAS c501chemistry analyzer (Mannheim, German).

### Survival Assays of HLBL-0102 Mice Treated with Cantide or 5-FU

Mice were treated with drug for 20 days as described above and observed until they were moribund. The life span of each animal was recorded and the percentage of prolonged life span was calculated as:

Prolonged rate (%) = *(L*_treat_/*L*_control_−1) ×100 where *L*_treat_ is life span of the treated group and *L*_control_ is life span of the non-treated group. Two independent experiments were performed on a total of 96 mice (48 mice per experiment).

### Statistical Analysis

Data were presented as mean ± standard deviation (SD) and tested with ANOVA unless otherwise stated. Group effects were adjusted using Bonferroni’s method. Survival was evaluated using the Log-rank method. LDH level was predicted by tumor volume using simple linear regression. Pearson’s correlation was applied to measure the relationship. Data were analyzed using SPSS 15.0 (SPSS, Inc., Chicago, IL, USA). A P-value <0.05 was considered statistically significant.

## Results

### Establishment and Characterization of HLBL-0102 Xenografts

A surgical specimen was obtained from the tumor mass in the left lobe of the liver of a patient diagnosed with hepatic primary lymphoma (non-Hodgkin’s B cell lymphoma) by Ann Arbor classification of IEA. Freshly obtained tumor fragments from the patient were surgically implanted into the left lobe of the liver in five nude mice for the first passage. We observed tumor growth in all the animals after a latency period of 7–45 days. The primary lymphoma of the liver, termed HLBL-0102, has currently been maintained for 42 generations (more than 3 years) by orthotopic passage (liver to liver) in nude mice and exhibited 100% transplantability. A total of 320 nude mice were implanted. The latency of tumors (time from implantation until rapid tumor growth) showed a significant decrease (median of 8.9 days) until generation 15, after which the growth rate was stabilized (Figure 1A). The survival time of the mice also decreased during the course of passaging (Figure 1B). The average passage interval (time between two implantations) was 27.4 days.

**Figure 3:**
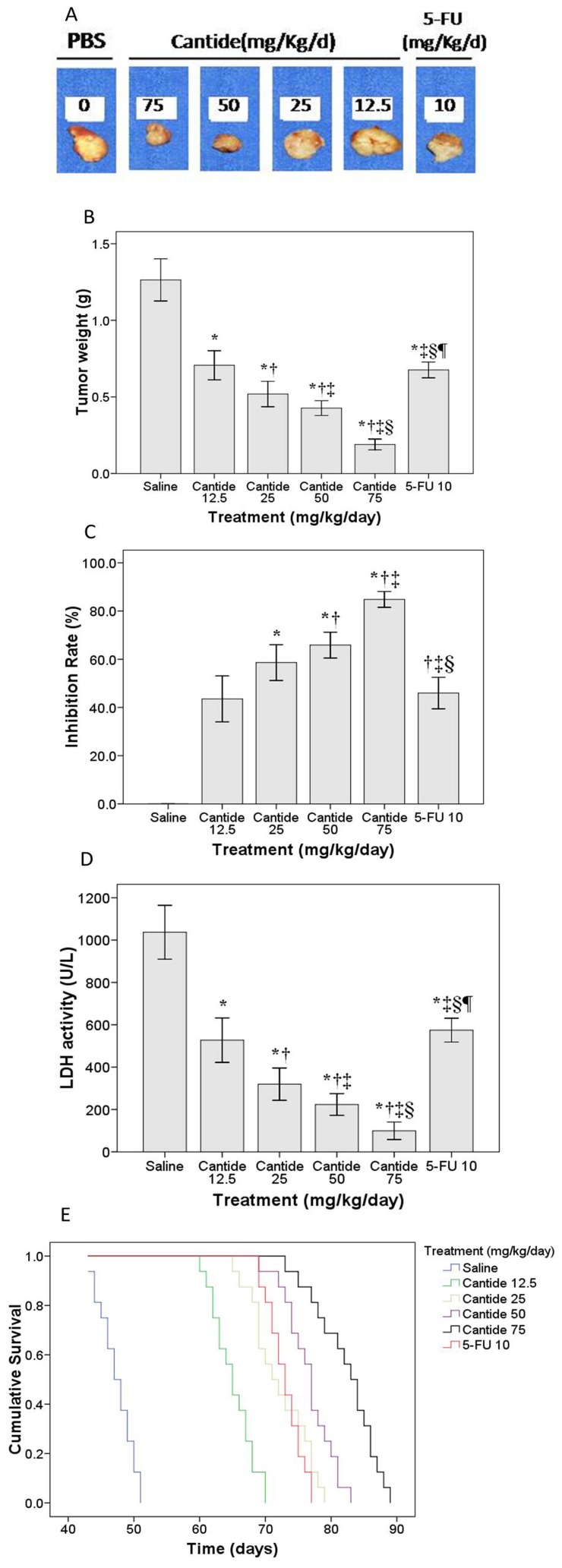
Effect of Cantide treatments on growth of HLBL-0102 PHL. (A) Representative images of PHL treated with different concentrations of Cantide for 20 days. 5-FU treatment was used as positive control and PBS treatment was used as a negative control. (B) Dose dependent decrease in tumor weight of PHL in cantide-treated HLBL-0102 mice. *P<0.05 compared to saline group, †*P*<0.05 compared to Cantide 12.5, ‡*P*<0.05 compared to Cantide 25, §*P*<0.05 compared to Cantide 50, ¶*P*<0.05 compared to Cantide 75. Mean ± standard deviation (n = 8 each group), Experiment repeated three times; all data adjusted using Bonferroni’smethod. (C) Tumor inhibition rate of PHL in Cantide-treated HLBL-0102 mice. *P<0.05 compared to Cantide 12.5, †*P*<0.05 compared to Cantide 25, ‡*P*<0.05 compared to Cantide 50, §*P*<0.05 compared to Cantide 75. Mean ± standard deviation, (n = 8 each group). Experiment repeated three times. All data adjusted using Bonferroni’s method**.** (D) Determination of serum LDH activity in Cantide-treated HLBL-0102 mice. There was a dose-dependent decrease in LDH activity in Cantide-treated mice. *P<0.05 compared to saline group, †*P*<0.05 compared to Cantide 12.5, ‡*P*<0.05 compared to Cantide 25, §*P*<0.05 compared to Cantide 50, ¶*P*<0.05 compared to Cantide 75. Mean ± standard deviation (n = 8 each group). Experiment repeated three times. All data adjusted with Bonferroni’s method. (**E**) Analysis of survival curves in Cantide-treated HLBL-0102 mice. Kaplan-Meier survival curves of HLBL-0102 mice treated with different concentrations of Cantide. Mice were injected with different doses of Cantide three days after establishing tumor xenografts as described in the Methods section. Treatment was continued for 20 days and mice were observed until moribund.

Figure 2A shows a representative image of a mouse with PHL in the 37^th^ generation of transplantation. We observed tumor growth in the livers of all the transplanted mice. Histological examination showed diffuse infiltration of liver tissue and breakage of the hepatic cord by lymphoma cells (Figure 2B). The ability of the implanted PHL in metastasis was also evaluated and the results suggested the lymphoma has limited metastatic activity that only a minority of these implanted mice (20%) had infiltrations to portal lymph nodes but not to the distal lymph nodes (data not shown), consistent with the notion that most PHLs are stationary. To verify the identity of the tumor implanted, we used immunohistochemistry to evaluate the presence of B cell lymphoma markers CD20 and CD79a. HLBL0102 tumor cells were strongly positive for both markers (Figures 2C and 2D), confirming the identity of the tumor. To further characterize the subtype of the B cell lymphoma in study, we used antibodies against subtype markers, Bcl-6, CD10 and IRF4 for immunohistochemistries. The results shows the B-cell lymphoma in study has Bcl-6 negative, CD10 negative and IRF4 positive phenotype, suggesting the lymphoma in study is a non-germinal center B-cell-like diffuse large B cell lymphoma.

### DNA Content Analysis by Flow Cytometry

There were significant differences in the DNA index and proliferation index between normal liver cells and PHL cells from HLBL-0102 mice (Table 1). Lymphoma cells from HLBL-0102 mice and lymphoma cells from the original patient were comparable with respect to DNA index and proliferation index (1.7±0.2 vs. 1.7±0.2 and 34.7±2.2 vs. 34.2±1.6). However, both indices were significantly higher in PHL from HLBL-0102 mice when compared to normal liver cells (1.7±0.2 vs. 1.0±0.1 and 34.7±2.2 vs. 14.7±1.4 respectively), suggesting a higher mitotic rate in HLBL-0102 primary lymphoma cells compared to liver cells. Similar to the original lymphoma cells from the patient, PHL cells from HLBL-0102 mice were aneuploid, indicating that cytogenetic characteristics were preserved.

### Cantide Inhibits Growth of HLBL-0102 Tumors

We evaluated the anti-tumor effect of Cantide on HLBL-0102 tumor xenografts *in vivo*. Starting on day 3 after the mice were transplanted orthotopically with 1-mm^3^ of HLBL-0102 tumor fragments, they were treated intravenously with different doses of Cantide, 5-FU (10 mg/kg/d) or physiological saline daily for 20 days. Tumors from mice under different conditions were dissected and tumor weights, growth inhibition rate were evaluated. The results showed a significant, dose-dependent inhibition of tumor growth in mice treated with 25, 50 and 75 mg/kg/d Cantide when compared to mice treated with saline. The highest inhibitory efficacy was achieved with 75 mg/kg/d of Cantide when compared to the saline-treated group or the 5-FU treated group (Figures 3A, B). This was paralleled by a corresponding, dose-dependent increase in the tumor inhibition rate in mice treated with increasing concentrations of Cantide (Figure 3C).

There was no significant difference in the mean body weight of mice in the different treatment groups (data not shown).

### Cantide Inhibits Plasma LDH Activity

The therapeutic efficacy of Cantide on mice with PHL was also evaluated by LDH activity, a well-established lymphoid tumor marker. We showed a significant dose-dependent decrease of LDH activity in the Cantide-treated animals compared to saline treated animals. The therapeutic efficacy of 5-FU(10 mg/kg/d) is similar to 12.5 mg/kg/d of Cantide, but significantly lower than the other three Cantide treatment groups (Figure 3D).

### Cantide Treatment Prolongs Survival in the HLBL-0102 Model

We evaluated the effect of Cantide on the life span of tumor-bearing mice. Tumor bearing mice treated with either 5-FU (10 mg/kg/d), saline or increasing doses of Cantide (12.5, 25, 50 or 75 mg/kg/d), were followed for a period of 90 days. We used Kaplan Maier survival curves to show that mice treated with different doses of Cantide survived significantly longer than saline-treated mice (Figure 3E). The effect of Cantide on life span extension was dose-dependent and Cantide treatment at a dose of 75 mg/kg/d resulted in the highest prolongation of life span (139%). Interestingly, mice treated with 75 mg/kg/d of Cantide survived significantly longer than mice treated with 10 mg/kg/d of 5-FU.

## Discussion

In this study, we used surgical orthotopic transplantation to establish a nude mouse model of PHL. We successfully performed orthotopic transfer of the PHL for 42 generations and carried out detailed characterizations of the tumor. Immunohistochemical analysis demonstrated that the tumor cells were positive for B cell lymphoma markers, CD20 and CD79a. The cytogenetic and mitotic characteristics of the original lymphoma from the patient were preserved in HLBL-0102 cells. We also evaluated the effects of Cantide, an antisense phosphorothioate oligonucleotide against hTERT, on the growth of HLBL-0102 tumors. We showed a significant, dose-dependent inhibition of tumor weight, tumor volume as well as a reduction in serum LDH level in the orthotopically transplanted animals. Importantly, survival was prolonged in HLBL-0102 tumor-bearing mice treated by Cantide when compared to untreated mice.

Although tumors implanted subcutaneously into nude mice have been shown to closely resemble the morphology and biochemistry of the original tumors, they rarely metastasize [31].This limitation was overcome by implanting the tumor cells or histologically intact tumor tissue into the anatomically correct site in nude mice [1]. Orthotopic mouse models using histologically intact human liver cancer specimens, were previously reported to closely mimic the natural progression of the disease in HCC patients in terms of tumorigenicity, metastasis and antigenic phenotype [9]. In our present study, we showed for the first time that orthotopically transplanted PHL, exhibited the antigenic phenotype of B cell lymphoma cells, exhibited 100% transplantability and showed autonomic and invasive growth. The tumor cells also preserved cytogenetic and mitotic characteristics of original primary hepatic lymophoma that are distinct from liver cells. Interestingly, we observed a gradual decrease in the latency time of the HLBL-0102 mice during the first 15 passages. We speculate that this could be because adaptation of the human lymphoma in mouse liver occurs over the first 15 passages after which the growth rate did not change, suggesting the generation of a stabilized xenograft line.

A number of antisense gene silencing strategies have been designed over the years, in which specific nucleic acid complementary to the sequence of target gene mRNA resulting in downregulation of the target gene [22]. Based on the fact that anti-apoptotic proteins, Bcl-2 and Bcl-xl, are upregulated in a number of tumors, antisense oligonucleotides directed to the mRNAs of these proteins have been therapeutically used in many cancers [24], [25], [32], [33]. Survivin, another anti-apoptotic protein, which is expressed during embryonic development in a cell cycle-regulated manner, has also been used as a target in lung cancer treatment [26].

Telomerase is a ribonucleoprotein enzyme complex which is activated in almost 90% of all human cancers [34]. Telomerase activity has been inhibited in different cancers using a number of strategies including antisense techniques, ribozymes against hTERT and introduction of a dominant negative form of hTERT into cancer cells [34]. The antitumor effect of Cantide was reported to be mediated via caspase-dependent apoptosis [27]. Lin et al found that inhibiting telomerase activity with Cantide destabilized telomeres, inhibited cell growth, resulted in cell death, and decreased the invasiveness and metastasis of HCC tumors in an orthotopic mouse model [30]. In this study, we showed for the first time that Cantide inhibited the growth of orthotopically transplanted PHL tumors. In agreement with the work of Lin et al. on HCC tumors, we also found a significant, dose-dependent inhibition of the weight and volume of PHL tumors in Cantide-treated mice when compared to saline-treated mice. Additionally, our work also found that Cantide treatment prolonged the survival of the HLBL-0102 mice and decreased their LDH activity, which is a lymphoid tumor marker. Our data, which are consistent with previous research, provide the basis for the clinical development of Cantide as a therapeutic agent for PHL.

One limitation of our present study is that we did not investigate the mechanisms underlying Cantide-mediated inhibition of PHL tumors. Based on the previous report that Cantide exerts its antitumor activity by inducing apoptosis via a caspase-dependent mechanism [27], we would like to evaluate the effect of Cantide on cell cycle-related events and on apoptosis in the HLBL-0102 model. Though Lin et al. combined Cantide with 5-FU and found that Cantide sensitized HCC tumors to 5-FU [30], we did not combine Cantide with 5-FU to determine if the sensitization occurred with PHL tumors. Antisense oligonucleotides to hTERT were also shown to sensitize human leukemia cells to cisplatin [35]. We would like to compare the effects of combining Cantide treatments with other chemotherapeutic agents versus using Cantide as a single agent. Given the change in growth rate of the xenograft during passaging, it will be interesting to explore if Cantide exerts differential activity in the early versus late passages of the xenograft.

In summary, we established an orthotopic mouse model of PHL in nude mice (HLBL-0102) and have orthotopically passaged the tumor for 42 generations to date. We showed that treatment of HLBL-0102 mice with antisense oligonucleotide targeted to hTERT (Cantide), resulted in a significant inhibition of tumor growth and increased survival of the treated mice. Our study could serve as the basis for the development of a clinical trial protocol to treat PHL.
